# Patent Foramen Ovale in Cerebral Infarction

**DOI:** 10.2174/157340310791658794

**Published:** 2010-08

**Authors:** J Serena, M. Jiménez-Nieto, Y. Silva, M. Castellanos

**Affiliations:** Department of Neurology and Stroke Research Unit. Institut d’Investigaciò Biomèdica de Girona, Spain

**Keywords:** Patent foramen ovale, right-to-left shunt, cryptogenic stroke, transcranial Doppler, echocardiography.

## Abstract

Recent studies support the hypothesis of a close aetiological and pathogenic association between the presence of patent foramen ovale (PFO) and cryptogenic stroke. The therapeutic options currently used in the treatment of these patients range from standard antiaggregation and standard-dose anticoagulation to the percutaneous occlusion of the PFO. The use or recommendation of treatment is based both on clinical risk factors associated with PFO, such as age, detection of states of hypercoagulability and previous history of stroke, and on the risks associated to right-to-left shunt (RLSh) and PFO, such as the size of PFO, magnitude of RLSh and the presence of atrial septal aneurysm (ASA). However, there is currently no consensus regarding the most suitable treatment and it is surprising to observe the widespread use of certain therapeutic approaches which are not supported by clinical evidence.

In this revision, we analyse the relevance of PFO in cryptogenic stroke, consider the main evidence available for determining the best management of these patients and make diagnostic and therapeutic management recommendations.

## INTRODUCTION

Between 30 and 40% of ischaemic strokes are of undetermined aetiology even after adequate study and this percentage is even higher in the case of stroke in young patients [1]. The presence of patent foramen ovale (PFO) has been suggested in numerous studies as a potential cause of paradoxical embolism and, in particular, of cerebral emboli in stroke of unknown origin [2-21] (Table **I**). Some of these studies have shown an association between the size of PFO, the magnitude of right-to-left shunt (RLSh), and the presence of atrial septal aneurysm (ASA) with increased stroke risk [22]. However, few studies have analysed the risk of recurrence and, particularly, the risk of recurrence in relation to factors involved in a first stroke and, as such, the best treatment for patients with PFO has yet to be established. This article aims to review the involvement of PFO in crypto-genic stroke and its relevance in stroke recurrence, and to reflect upon the current therapeutic options for patients with this anomaly.

## BACKGROUND

**Fig. (1):**
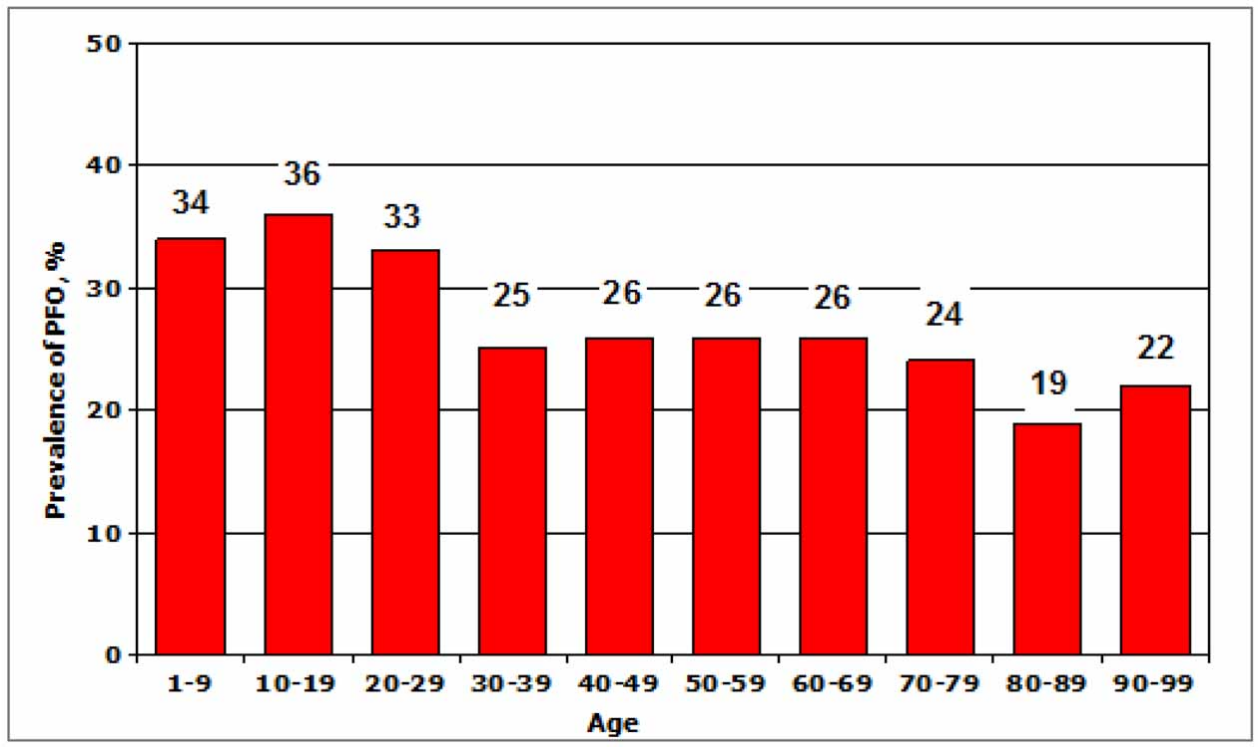
Prevalence of PFO in autopsy studies in subjects without heart disease.

Paradoxical embolism from the venous to the arterial systems through RLSh may be secondary to the presence of atrial septal or ventricular defects, pulmonary arteriovenous malformations, and, fundamentally, to the existence of a PFO. The PFO is a channel that is open during the foetal period and is the normal pathway of venous blood from the right to left cardiac cavities, before heading on, avoiding the pulmonary bed, towards the placenta, where it is oxygenated. After birth, the PFO is sealed functionally due to there being greater pressure in the left atrium than in the right atrium and so the venous blood now heads from the right atrium to the pulmonary bed and, after crossing this filter, moves on to the left atrium. Permanent closure, by the adhesion of the septum primum to the septum secundum, typically occurs in the first three months after birth. However, there is a large group of people for whom the foramen ovale remains permeable. PFO is detected in 27-35% of normal hearts in autopsy studies [23,24] (Fig. **1**), in 10-26% of healthy individuals by transoesophageal echocardiography with contrast (c-TEE) and in 25-35% through transcranial Doppler with contrast (c-TCD). The diameter of the PFO in autopsy studies in normal hearts oscillates between 1 and 19 mm, with a mean of 4.9 mm. The pathological relevance of these apparently inconsequential diameters becomes clear when we consider that an embolus of just 1 mm is sufficient to occlude a large cortical arterial branch and that an embolus of 3 mm is sufficient to occlude the middle cerebral artery causing a massive hemispheric infarction.

Paradoxical embolism was first described by Cohnheim in 1877 [25]. In 1972, by which time just 128 cases had been described worldwide, four necessary criteria were proposed for the acceptance of paradoxical embolism. These included the demonstration of venous thrombosis or of pulmonary embolism together with an increase in the pressure in the right cardiac cavities. In 1988 two studies were published which led to a reevaluation of the importance of PFO [2,3]. Both used contrast echocardiography in finding a 40-50% prevalence of PFO in cryptogenic stroke in young patients in comparison with 10-15% in healthy controls. Since then, many studies have confirmed the close association between PFO and ischaemic stroke (Table **I**).

Although paradoxical embolism, which is the passage of a thrombus from the peripheral venous system to the left cardiac cavities through the PFO, seems to be the most likely mechanism in patients with cryptogenic stroke associated to PFO, other potential mechanisms should also be considered. One proposed mechanism is in situ thrombus formation within the foramen ovale channel itself. Alternatively, patients with PFO may be more susceptible to atrial fibrillation, due to an altered atrial electrical substrate [26]. Finally, we need to consider the possibility that patent foramen ovale discovered in patients with cryptogenic stroke might be incidental as has been argued to be the case by Kent D. *et al*. in one third of patients by applying Bayes’ theorem [27]. 

## HOW TO DETECT PFO USING CONTRAST ECHOCARDIOGRAPHY AND TRANSCRANIAL DOPPLER 

PFO is detected by showing the passage of air microbubbles from the right to the left cardiac cavities after the endovenous administration of echo contrast. Although there are commercially available echo contrasts, the technique using saline contrast has been validated and has the advantages of being inexpensive, safe and available. Detection of RLSh through a PFO can be performed with c-TTE, c-TEE and c-TCD. As c-TEE detects approximately twice as many PFO’s as c-TTE, it is considered as being the diagnostic reference test. 

### TEE Protocol

As part of the routine aetiological study of a patient who has suffered an ischaemic stroke, patients should undergo a complete transthoracic study before TEE evaluation. In general, TEE study is performed after topical anaesthesia of the oropharynx and mild sedation with intravenous midazolam (0.5 to 1.5 mg). The echoscope is entered into the oesophagus, and the tip is tilted to permit a clear image of the atrial septum at the level of the fossa ovalis. Contrast material is prepared and injected into the antecubital vein as described in the TCD protocol below. The contrast examination is performed by studying the horizontal and the longitudinal views with the patient breathing normally and during a Valsalva manoeuvre. A PFO is diagnosed when microbubbles are detected in the left atrium within three cardiac cycles of their appearance in the right atrium. On the other hand, when microbubbles appear after the third beat these should normally be attributed to intrapulmonary shunt. Although there is no consensus as how to classify RLSh by c-TEE, three groups are often accepted: small (less than10 microbubbles); moderate, when too many microbubbles appear in the left atrium to be counted, but without being echogenic as in the right atrium; and, finally, severe, when microbubbles caused echogenicity with at least the same intensity in a part of the left atrium as in the right atrium [21]. Before examination with contrast, all patients undergo a complete TEE study to evaluate the size of the left atrium, the left atrial appendage, the presence of atrial spontaneous contrast and/or thrombi, atrial septum aneurysm, interatrial septum defect, and other potential sources of cardiac embolism. Finally, the probe is advanced 40 cm from the incisors, rotated through 180°, and slowly withdrawn to examine the descending thoracic aorta and the aortic arch for atherosclerotic plaques or thrombi as possible sources of embolization. 

Although second harmonic imaging has increased TTE sensitivity to between 63% and 100% [28-30], c-TEE remains the standard echocardiographic technique, particularly in young patients suffering from cryptogenic stroke.

### TCD Protocol

**Fig. (2):**
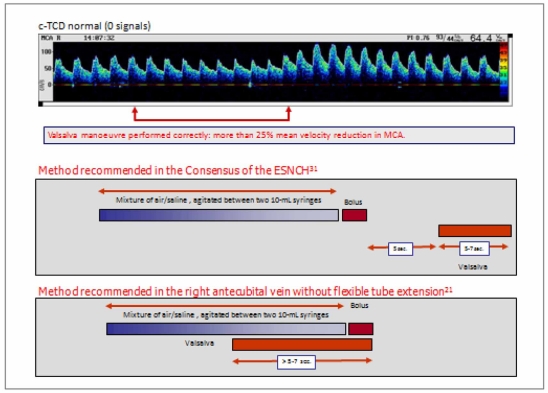
Summary of the RLSh detection methodology using TCD.

**Fig. (3):**
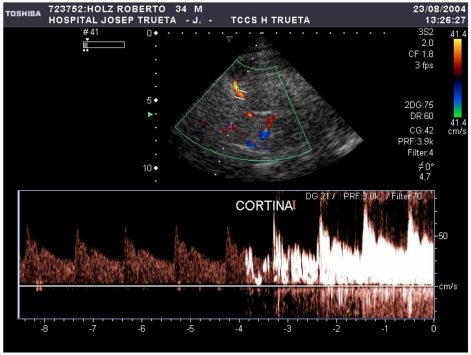
Massive RLSh by transcranial colour coded duplex sonography detected 3 seconds after contrast infusion (agitated saline solution, 9 mL of saline solution and 1 mL of air). Video can be downloaded at http://www.telefonica.net/web2/jserenal/pulmonaryshunt/fistula.htm

TCD examination may be performed either by using TCD or transcranial colour-coded duplex sonography. Although there tends not to be significant differences between the number of signals detected in the right and left middle cerebral arteries (MCA), TCD permits the simultaneous monitoring of both MCA‘s through the temporal window by the use of 2-MHz probes. For bilateral monitoring, TCD probes can be fitted using either a headband or light metal frame that allows the probes to be fixed firmly to the head. The contrast for the study is usually obtained by a mixture of sterile saline solution (9 mL) and air (1 mL), agitated between two 10-mL syringes, connected by a three-way stopcock. The solution should be immediately injected with a 20-gauge/32-mm catheter placed in the antecubital vein to obtain a bolus of air microbubbles. This procedure should be performed during normal breathing and afterwards during a Valsalva manoeuvre, usually three times. The Valsalva manoeuvre can be standardized by asking the subjects to blow into a manometer until 50 to 60 mm Hg of pressure is reached and to maintain this pressure for a period of at least five to seven seconds. The bolus of air microbubbles should be injected during Valsalva manoeuvre in one to two seconds at the end of this seven second period (Fig. **2**). Subjects are previously instructed in the performance of the Valsalva manoeuvre, the efficacy of which can be gauged by a reduction of at least 25% in the mean velocity of the MCA. The consensus reached at the 4th Meeting of the European Society of Neurosonology and Cerebral Hemodynamics (ESNCH) [31] agreed that a time window from contrast infusion to signal detection in MCA cannot differentiate intracardiac and extracardiac shunt. From an aetiopathogenic point of view, an extracardiac shunt has the same relevance as an intracardiac shunt as a potential cause of stroke. RLSh should be suspected as being due to PFO when it is greater during the Valsalva manoeuvre than at rest whereas in extracardiac shunt the magnitude tends to be of a similar magnitude, and normally of longer duration, in the two states [32]. The most frequent extracardiac shunt, the arteriovenous pulmonary fistula, associated to an 18% incidence of stroke, is often clinically silent and can easily be suspected by c-TCD due to the specific profile of the RLSh when compared with that of the PFO [32-34] (Fig. **3** and web page video). As can be seen in Fig. **3** and the video, it only takes three seconds for massive RLSh after contrast infusion in basal conditions [34].

**Fig. (4):**
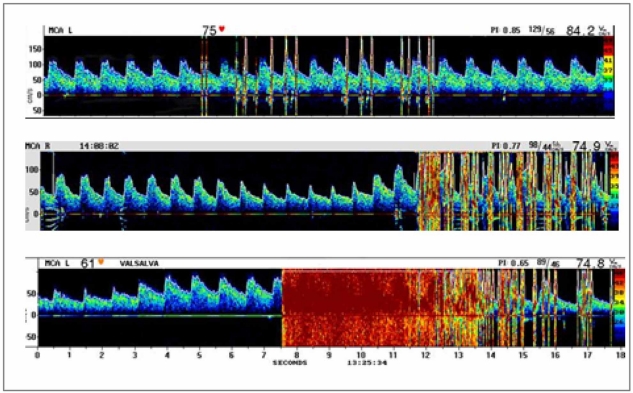
Contrast TCD detected RLSh of less than 10 signals (upper panel), “shower” (middle panel), and “curtain” (lower panel) patterns in MCA after Valsalva manoeuvre.

Whether intracardiac or extracardiac, the relevance of RLSh depends on its magnitude, which is determined by counting the number of signals in the MCA. Patients are usually divided into 3 different groups on the basis of the maximum number of microbubble signals in the MCA in any single frame after intravenous injection of agitated saline solution: “normal” TCD study (if 0 signals were detected), “small” RLSh (10 signals), and “large” degree of shunt (more than 10 signals). In this last group, “shower” (more than 25 microbubbles) and “curtain” (uncountable microbubbles) are particularly relevant and have been associated with cryptogenic stroke in case-control studies [21] (Fig. **4**).

There remains some discussion with regards to what the most appropriate methodology is to increase the sensitivity of RLSh detection using Valsalva manoeuvre and TCD. The consensus of the ESNCH recommended that Valsalva manoeuvre should be started 5 seconds after the beginning of the contrast injection. A comparison between different injection modes found increased sensitivity using injection before Valsalva manoeuvre with air/saline as a contrast agent. This is particularly true when a distal vein is used or when a short flexible tube is added to the needle in the antecubital vein. However, when the antecubital vein is used in the right arm without extension, a Valsalva manoeuvre and simultaneous contrast infusion increases the sensitivity of RLSh detection (Fig. **2**).

**Fig. (5):**
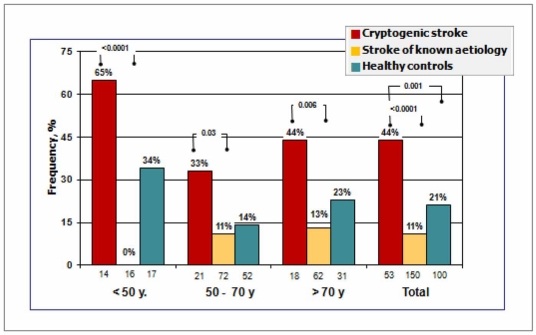
Frequency of massive RLSh during Valsalva manoeuvre by age group in cryptogenic stroke, stroke of known aetiology, and in healthy controls. Number of patients in each group are given along the horizontal axis. Probability values representing the differences between groups are shown at the top of the bars.

Despite several studies having demonstrated that c-TCD has a sensitivity and specificity which is equal to or superior to c-TEE, this latter technique continues to be considered as the diagnostic test of reference given that it permits the direct visualisation of the size of the PFO and the demonstration of additional cardiac sources of cerebral embolism such as the presence of septal aneurism and atherosclerosis of the aortic arch. However, it should be noted that c–TEE has certain limitations in the detection of RLSH: 10% of patients do not tolerate the performance of the test, the Valsalva manoeuvre is difficult to perform and standardise as TEE is a semi-invasive technique often requiring patients to be sedated, is problematic for patients with stroke and swallowing difficulties, and, given that it is semi-invasive, is not suitable for systematic use or in control populations. In recent years, published studies have shown that in comparison with c-TEE, c-TCD is more sensitive both in the detection and quantification of RLSh. Furthermore, c-TCD has the advantages of studying the repercussion of RLSh directly in the target organ and of being easier to use, which makes it ideal both as a diagnostic test and for follow-up [7-11,21,31,35]. The prevalence of RLSh found using c-TCD in healthy controls (32%) is similar to the prevalence of PFO in autopsy studies [23,24], which lends support to the precision of c-TCD in the detection of RLSh. Although the prevalence of RLSh is similar in ischaemic stroke and healthy populations, the prevalence is particularly high in the subgroup of young patients with a stroke of unknown origin [2,3,6,9,16,17] and, as we have recently been able to show, in patients of any age (Fig. **5**) [21]. 

In the evaluation of the magnitude of RLSh it is essential to perform the Valsalva manoeuvre correctly. In early studies, c-TEE and c-TCD were carried our simultaneously and so incorrect or insufficient Valsalva manoeuvre affected both techniques. c-TCD is better than c-TEE in the detection and quantification of the magnitude of RLSh when both techniques are conducted independently both during the manoeuvre and in basal conditions. The most important limitation of c-TCD is the absence of a temporal bone window in 10% of patients who suffer stroke, a fact which particularly affects the older population although even in these cases the existence or not of RLSh can be established by insonating the internal carotid artery, the vertebral arteries or the basilar artery. 

In the specific case of cryptogenic stroke, the usefulness of TEE is not so much in the detection of PFO as in the demonstration of other associated cardioembolic sources and in particular the existence of atrial septal aneurysm (ASA), which associated with PFO increases the risk of stroke through PFO or isolated ASA. In the opinion of the present authors, c-TCD is an important part of the ultrasonographic study that should be performed in patients with cryptogenic stroke particularly in young patients when paradoxical embolism should be suspected. The aetiological study of stroke must be accompanied by TTE or TEE.

## POTENTIAL MARKERS OF STROKE RECURRENCE

**Fig. (6):**
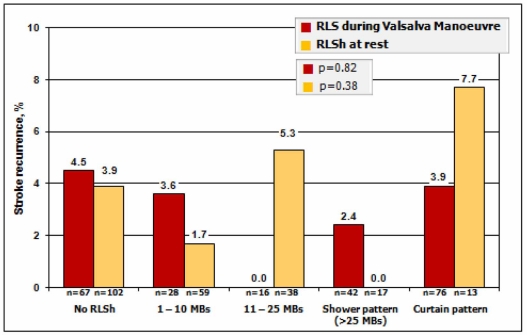
CODICIA Study. Stroke recurrence by RLSh magnitude at rest and during Valsalva manoeuvre in the younger population.

The presence of RLSh is in itself an inadequate factor for the prediction of stroke risk due to the fact that 30% of the healthy population share this condition. Several studies have analysed the characteristics associated with a greater risk of ischaemic stroke: the size of the PFO^20^ and, especially, the magnitude of the shunt have been found to increase risk. An average of 13.9 microbubbles is detected in the left atrium in patients with cryptogenic stroke as compared to 1.6 in patients with a known cause of stroke. De Castro *et al*. suggested the importance of RLSh magnitude in PFO on detecting those patients with PFO and cryptogenic stroke with ischaemic lesions in cranial CT showed a greater number of microbubbles in the left atrium than patients without cranial CT lesions. The c-TCD study has shown itself to be an efficient tool in the quantification of the shunt as was recognised by the previously mentioned Consensus Conference of the ESNCH [31]. Although the aetiopathogenic and therapeutic importance of the magnitude of shunt has not been fully established, the available results highlight the crucial importance of its quantification. Traditionally the importance of quantifying the magnitude of RLSh during the Valsalva manoeuvre is emphasised [18], however this is an aspect which could also be questioned. The magnitude of RLSh is typically higher with Valsalva manoeuvre than at rest but the presence of RLSh at rest may be of more clinically relevance. Patients with a shunt during quiet breathing have an increased time exposure to paradoxical embolism and should in theory be at increased risk of stroke recurrence. De Castro *et al*. [36] reported that the cumulative risk of cerebrovascular event recurrence at three years was higher (12.5%) in high risk PFO, defined as a mobile septum, and RLSh at rest than low risk PFO defined as RLSh only with Valsalva manoeuvre (event rate of 4.3%). A similar finding is suggested by a post-hoc analysis of the CODICIA study [37]. Stroke recurrence event rates, based on the RLSh magnitude groups defined by the maximum number of microbubble signals both during Valsalva manoeuvre and at rest in the whole population (n=486) and in the younger group (n=229), are presented in Fig. **6**. Although no significant differences were obtained at rest in comparison with the main results, an increased risk of stroke recurrence was detected when only the curtain pattern at rest was considered. 

However, the common identification of PFO in patients with established causes of stroke (atrial fibrillation, carotid stenosis) and the absence of factors which are traditionally associated with paradoxical embolism in patients with PFO and stroke (such as a previous history of thrombophlebitis; clinical criteria, ECG or echocardiographs showing pulmonary hypertension; and especially a stroke onset associated with Valsalva manoeuvres increasing the pressure in the right cardiac cavities such as coughing or overexertion) have called into question the aetiopathogenic role of PFO in stroke. This is particularly the case in patients of advanced age, which is the population with the most frequent stroke risk and with the greatest number of associated vascular risk factors. In any case, although somewhat difficult to interpret, some studies have shown an association between PFO and cryptogenic stroke in older populations of patients (>55 y) [21,38]. PFO remained strongly associated with cryptogenic stroke after adjustment for stroke risk factors and so should be considered as a potential cause of stroke as the figures are similar to those found in younger patients (Fig. **5**).

Amongst the potential anomalies associated to PFO detected by TEE that might explain increased stroke risk are ASA, Chiari networks (CN) and a prominent Eustachian valve (EV). Although there is no consensus as to the definition of ASA, it is generally accepted as being an excursion of the interatrial septum into either atrial cavity of at least 15 mm [39]. In fact, PFO and its magnitude is a consequence of the presence of ASA. While the prevalence of ASA in the healthy population is only 1% [40], its prevalence in patients with massive RLSh reaches 30-40%.

Persistent EV or CN in adulthood has been suggested as one of the causes of RLSh in the absence of pulmonary hypertension [41,42]. The EV, which is derived from the right sinus venosus valve, has a semicircular shape and faces the anterior-inferior surface of the inferior vena cava to the atrial septum. CN, which have been found in 1.3% to 4% of autopsies, represent a large multi-perforated Eustachian valve with a network-like appearance. The EV and CN direct the flow of the inferior vena cava towards the foramen ovale during foetal life leading, in the case that it persists, to an adult predisposition to paradoxical embolism [22,43]. Large PFO and prominent EV or right atrial filamentous strands have been found more frequently in patients with ASA than those without (37.7% vs. 10.9%, p < 0.001 and 59.4% vs. 43.1%, p = 0.02). In TEE studies, an EV is present in 48% of patients with cryptogenic stroke [44] and large CN are associated with PFO in 83% of cases [45]. 

Finally, prothrombotic states may lay behind paradoxical embolization through a PFO. Most studies have failed to find an association between cryptogenic stroke, PFO and prothrombotic diseases. However, recently factor V Leiden and prothrombin G20210A mutations have been described as being significantly more prevalent in patients with cryptogenic stroke and PFO than in matched healthy controls (11% versus 2%; 95% CI, 0.04 to 0.94; p<0.05) or cryptogenic stroke without PFO (11% versus 1.1%; 95% CI, 1.09 to 109; P<0.05) [46].

## RISK OF STROKE RECURRENCE IN CRYPTOGENIC ASSOCIATED WITH PFO

Although the association between PFO and cryptogenic stroke has been well established by many case-control studies [2-21] and the metaanalysis published by Overell *et al*. [22] confirms a consistent association in young patients (<55 y) between cryptogenic stroke and PFO (OR 6.00, 95% CI 3.72-9.68) and particularly of PFO+ASA (OR 17.09, 95% CI 2.19-133.46) when compared with stroke of known cause or healthy control PFO, the very few prospective studies that have analyzed the importance of PFO as a predictor of stroke or recurrence have given variable and sometimes conflicting results [47-50]. Furthermore, only one of these studies performed a randomised analysis of the efficacy of medical treatment in the secondary prevention of stroke and none has compared medical treatment with percutaneous occlusion in a prospective and randomised study (Table **II**). 

The conclusions we can draw from the results of published prospective studies in the literature are complementary to one another given that they have not been performed with a uniform design. The French study [48], which included only young patients (<55 years) with cryptogenic stroke, found an increase in the risk of recurrence in those patients with a combination of both PFO and ASA, independently of the size of the PFO, and no increase in the risk of patients with just either PFO or ASA. Despite the fact that there has been an ever more generalised tendency to close the PFO in young patients with PFO+ASA since the publication of this study, these results have not been confirmed by later studies (PICSS and CODICIA) and they were based on a subgroup of only 51 patients, who suffered six recurrences in four years. 

Similarly to the French study, the PICSS study [49] was performed using contrast TEE in the evaluation of PFO, although in this case it was not limited to the study of young patients. The PICSS study used the design of the WARSS clinical trial that had randomised patients with non-cardioembolic stroke in two branches of medical treatment (anticoagulation vs. antiplatelet) in an attempt to demonstrate that anticoagulation has greater efficacy in patients that habitually receive antiplatelet therapy. The PICSS study included 630 patients from a total of 2,206 randomized in the main study. Of these 630, 265 (42.1%) were classified as cryptogenic, 244 (38.7%) as lacunar, 68 (10.8%) large-vessel, 27 (4.3%) other determined cause, and 26 (4.1%) as conflicting mechanism. The results of this, the only randomised study did not show an increase in the risk of recurrence associated to PFO, whether with or without ASA, neither in patients with a known cause of stroke nor in the cryptogenic stroke group. The design of the PICSS study for the first time permitted a randomised evaluation of the efficacy of anticoagulant treatment in the prevention of recurrence and demonstrated that anticoagulation is not more efficient than antiplatelet therapy in the prevention of recurrence.

**Fig. (7):**
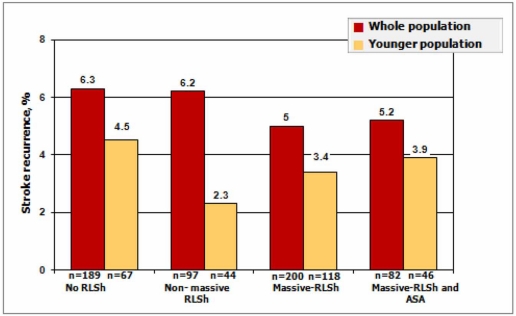
Stroke recurrence by RLSh magnitude during Valsalva manoeuvre and presence of ASA in the whole and younger populations in the CODICIA Study.

Finally, the Multicentre Study into RLSh in Cryptogenic Stroke (CODICIA Study) was a prospective, multicentre, observational study undertaken by the Cerebrovascular Diseases Group of the Spanish Neurological Society [50]. CODICIA included 486 patients older than 18 years with a recent cryptogenic ischaemic stroke. Functional outcome and stroke recurrence were evaluated at three months and yearly, during a mean time period of 729+/-411 days. The risk of recurrence in the CODICIA study was low and similar in patients with and without RLSh/PFO, independently of whether or not this was associated with ASA and whether the shunt was massive [21] (Fig. **7**). 

In addition to these four studies which had the objective of evaluating the risk of stroke recurrence, there are also two prospective studies that analysed the risk of a first stroke. The “Stroke Prevention: Assessment of Risk in a Community” (SPARC) study is the largest transoesophageal echocardiography-based study of stroke outcome in a random sample of a community [51] whereas the Northern Manhattan Study (NOMAS) have included the largest population using transthoracic 2-dimensional echocardiography [52] (Table **II**). These studies are particularly interesting to analyse given that, as we have commented, 30% of the healthy population have a PFO. Meissner *et al*. conducted a study that included c-TEE in a population of 585 healthy subjects older than 45 years who were participants in the SPARC study, which was conducted in the Olmsted County population (a community of 1,475 inhabitants) as part of the Rochester Epidemiology Project in Rochester, Minnesota. After being followed for five years, the annual incidence of ischaemic events was 1.19% (6.9% in 5.1 years). The presence of PFO and its size were not predictors of a first stroke (RR: 1.46, 95% 0.74-2.88, p=0.28) as neither was the presence of ASA (RR: 3.72, 95% 0.88-15.71, p=0.07). In this last case there was a tendency towards statistical significance but the figure is of just 11 patients out of a total of 581 (1.9%) and there was no risk data in patients with ASA+PFO. Although this study was conducted in a population with a relatively advanced age (>45 years, mean 66.9 +/- 13.3), the data were adjusted for the presence of classical vascular risk factors. The results obtained offer further support to there being a low risk of stroke in the PFO population and, hence, that primary prevention measures, invasive therapies such as percutaneous occlusion, and screening of particular risk groups is not at this time advisable. Similar results were found in the Northern Manhattan Study (NOMAS) [52] where PFO, whether alone or together with ASA, was not associated with an increased stroke risk in this multiethnic cohort. Although isolated ASA was associated with elevated stroke incidence, the authors advise that this result needs to be interpreted cautiously as again the sample is not representative (only eight patients out of a total sample of 1,100 who suffered two stroke recurrences in a mean time of follow-up of 6.6 years; HR 3.66, 95% CI 0.88 to 15.30, p=n.s.). 

In the analysis of recurrence in other studies, the high risk found in the PICSS study particularly stands out (7%/year in comparison with 2-3% in the rest of the prospective studies). This finding has been attributed to the greater prevalence of risk factors and the greater age of the patients in this study (59.0 +/- 12.2 years). The presence of PFO did not increase the risk of recurrence in cryptogenic stroke in young patients in the PICSS study whereas it did increase the risk in patients with an age of ≥ 65 years [53].

So, how can we explain the discrepancy between the consistent results of case-control studies and the often negative results in prospective ones? Differences in study design could offer a partial explanation. An additional one might be that treatment with antiplatelet agents or anticoagulant could be effective in decreasing the risk of stroke recurrence associated with PFO detected in case-control studies. On the other hand, a true increase in the risk of stroke recurrence associated to PFO might be counter-balanced by a similar increase in the risk associated to other factors in patients without PFO [50]. Finally, the average follow-up duration of the prospective studies may be too short to evaluate the true risk of stroke recurrence associated to PFO.

## THERAPEUTIC MANAGEMENT OF PATIENTS WITH PFO

There is agreement in not recommending any therapeutic primary prevention measures in subjects with PFO. However, the ideal treatment of patients who have suffered a cryptogenic stroke with PFO as a potential cause has not been established. The options range from classical antiplatelet treatment or anticoagulant therapy to surgical treatment.

Anticoagulant treatment in patients with cryptogenic stroke and PFO has become a frequent practice that has even been recommended in international conferences dealing with the subject. This has occurred particularly since the publication of the PFO-ASA French study in spite of the fact that the French study did not evaluate the efficacy of any particular treatment (all of the patients received antiplatelet therapy) and no therapeutic recommendation was made. Regrettably anticoagulation is still used for this type of patients despite the fact that the PICSS study, whilst showing an increase in risk, did not find this treatment to be more effective.

After the PICSS study, only the CODICIA study has analysed the efficacy of different treatments in a prospective fashion (Table **II**). CODICIA was not specifically designed to investigate the efficacy of one or other treatment since the most adequate treatment was left to the criteria of the clinician. However, the volume of patients included (n=486) together with the habit of anticoagulating patients with RLSh/PFO made a sufficient volume of patients available in each group as to permit an approximation to the efficacy of the medical treatment although the limitations inherent in the design need to be borne in mind. In the CODICIA study, 20.8% of patients received anticoagulant treatment for the prevention of recurrence. The conclusions of the CODICIA study are similar to the PICSS study: anticoagulant treatment is not significantly superior to antiplatelet therapy in the prevention of stroke recurrence. As is seen in Table **II**, there was a non-significant tendency to anticoagulation providing a greater benefit than antiplatelet therapy in the subgroup of patients of greater age with cryptogenic stroke but again there was no difference between patients with and without massive RLSh. Despite the tendency to a greater benefit of anticoagulation in older patients with cryptogenic stroke, which was also observed in the PICSS study, the results and design of the CODICIA, PICSS and WARSS studies do not justify its prolonged use at the present time, especially if we consider the secondary effects of warfarin and coumarin (risk: 2-3%/year, fatal 0.2%/year), which accumulate over time and are more serious than the risk of stroke recurrence due to the natural evolution of the disease. On the other hand, and as we have just commented, this benefit should be applicable equally to patients with cryptogenic stroke and PFO as to those who have cryptogenic stroke without PFO. 

Endovascular treatment, usually with the insertion of a double disk or an umbrella that allows the PFO to be closed, is a technique that is being used with great enthusiasm despite it not being without risks and the fact that materials have not yet been developed that avoid deterioration and loss of efficacy over time. As has been reported in many studies [54,55], PFO closure is efficient and relatively safe. The incidence of major and minor complications are 1.5% and 7.9% respectively. The annual rate of stroke is 1.98% (95% CI, 1.48 to 2.60) and the rate of stroke or death is 3.12% (95% CI, 2.32 to 4.11) in medically treated patients while in percutaneous closure of PFO these same figures are 0.19% (95% CI, 0.05 to 0.49) and 1.15% (95% CI, 0.46 to 2.37) [56,57]. Both metaanalyses comparing secondary prevention studies of transcatheter closure and medical therapy for PFO in cryptogenic stroke suggest the superiority of percutaneous occlusion in comparison with medical treatment. Khairy *et al*. conclude that percutaneous occlusion of PFO “may prevent a substantial proportion of cryptogenic strokes” whilst highlighting the need for randomised clinical trials [55]. However, the comparison of medical therapy with percutaneous closure is indirect as there are no results from clinical trials comparing medical and transcatheter closure treatment in a randomized setting and, in a recent collective analysis, there are no convincing data to indicate that the presence of PFO increases recurrent events in medically treated patients [57,58]. If we consider studies where PFO in cryptogenic stroke is compared with a non-PFO group [36,48-50,59], the relative risk of recurrent stroke or TIA ranges between 0.5 and 1.7, without statistically significant differences in the risk of recurrence in patient with and without PFO (RR 1.1, 95% CI 0.8 to 1.5; p=n.s.).

The key point would seem to be that percutaneous occlusion is probably the best therapeutic option if we are able to identify by clinical trials the precise risk group that would benefit from it. Meanwhile, the current evidence should advise against percutaneous occlusion of PFO other than in exceptional cases which should always be after a multidisciplinary evaluation of the patient and, in the opinion of the present authors, on being indicated by a neurologist who is an expert in vascular pathology. Having said this, Khairy’s metaanalysis should be interpreted with caution given the difficult task the authors faced in attempting to draw conclusions from such varied studies. The metaanalysis presents results derived from non-controlled studies, with different variables which were not previously defined, without homogeneity of the end-points, with highly variable methodologies in the detection of RLSh/PFO, and without safety committees nor follow-up of the registered cases. Furthermore, the basal characteristics of the patients included are not homogenous between the studies. The prevalence of classical stroke risk factors such as smoking habit, diabetes, advanced age and a greater proportion of males is significantly higher in those studies in which patients received a medical treatment, whilst in patients where percutaneous occlusion of the PFO was indicated, there were not only fewer classical risk factors but also a larger incidence of right-to-left shunt and subjects were more likely to have suffered more than one cerebrovascular event.

One aspect which has been little evaluated but which is very interesting when deciding which type of therapeutic intervention to undertake is the analysis of the consequences of suffering a stroke associated with RLSh/PFO. The fact that cryptogenic stroke associated with a RLSh presents a low annual risk of recurrence together with the lesser severity of stroke associated with RLSh shown by the CODICIA study [21,60] advises caution in the treatment selected for secondary prevention as, of course, we must not forget that any intervention must base itself on a risk/benefit analysis. As is shown in Fig. (**8**), stroke associated with RLSh/PFO has a better functional prognosis than cryptogenic stroke without RLSH/PFO. This is due to the lesser volume of the infarct in patients with stroke and RLSh in comparison with the volume of the infarct of cryptogenic stroke without RLSh (14.3 ml [1.5-35.4] vs. 6.5 ml [1.3-16.6]) and suggests that the mechanism of stroke in patients with and without RLSh/PFO is different. This hypothesis is supported by the greater prevalence of risk factors in patients with cryptogenic stroke without RLSh. The mechanisms of the stroke involved in RLSh appear less severe than those involved in patients without RLSh. We must be careful not to fall into the logical error of confusing the identification of a specific aetiology for a condition with the need to aggressively combat it. 

## FINAL RECOMMENDATIONS

Before indicating PFO closure, anticoagulation or antiplatelet therapy, we need to identify the subgroup of patients at high risk of stroke recurrence which may benefit from the application of these therapies. Several stroke associations, including the American Heart Association, American Stroke Association, American Academy of Neurology [58,61] and the European Stroke Organization (ESO) [62] recommend antiplatelet agents to prevent recurrent events (Class IIa, Level of Evidence B) whilst waiting for the results of ongoing clinical trials in PFO closure as there is currently insufficient data to make a recommendation. A further reason for a cautious approach at present is that better technology is currently being developed which aims to seal the PFO in such a way that either no device is left in place or that any device that is implanted is either partially or completely bioabsorbable. 

Anticoagulant therapy may be used in selected cases with high risk of thromboembolic events such as hypercoagulable states or evidence of deep venous thrombosis (Class IIa, Level of Evidence C). In clinical practice, PFO closure should be individualized and considered in young patients with recurrent stroke receiving medical treatment or in previously mentioned situations where anticoagulant treatment is considered (Class IIb, Level of Evidence C). Ongoing trials have a low recruitment rate with a risk of bias if younger patients or those with severe PFO or associated anomalies are treated out of the trials. It is therefore important that effort should be made to randomize patients systematically in these trials (www.ClinicalTrials.gov) and to improve the collaboration between neurologists, basic scientists and cardiologists so that reliable results regarding the best treatment options can be established for our patients as soon as possible.

## Figures and Tables

**Fig. (8):**
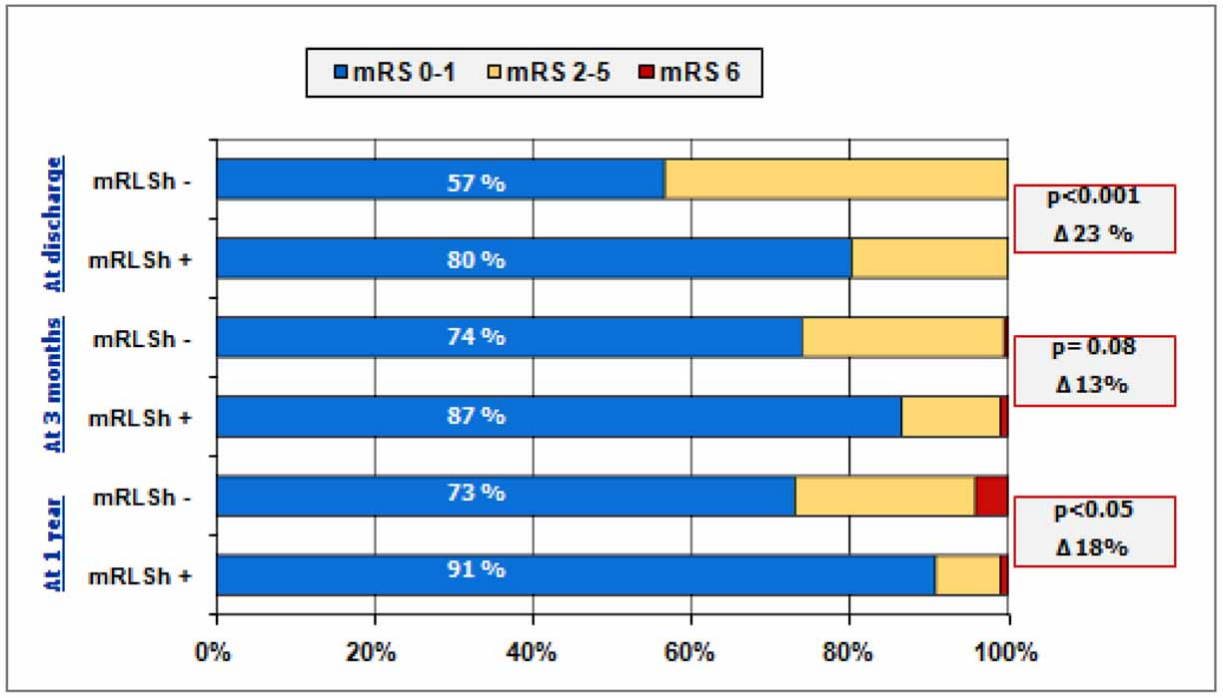
Functional outcome using the modified Rankin Scale (mRS) at discharge, 3 months and 1 year after cryptogenic stroke in patients with and without massive RLSh.

**Table I:** Prevalence of PFO by Echocardiography and/or c-TCD and Characteristics of Different Published Studies

Author	Year	Unselected patients	Age Range (mean)	N° of Patients with Stroke	% PFO patients c-TEE c-TCD	RLSh Quantification	% PFO in Cryptogenic Stroke	Controls n (% PFO)
Lechat *et al.* [2]	1988	No	<55 (36)	60	40* n.p.	No	54	100 (10)
Webster *et al.* [3]	1988	No	<44 (36.1)	40	50* n.p.	No	56	40 (15)
Pearson [18]	1991	No	17-84 (59)	79	11.3 n.p.	No	11.3	No
Hausmann [16]	1992	No	18-84 (52+/-10)	103	26.1 n.p	No	31.6	116 (21.6)
de Belder [17]	1992	No	16-84	104	21.1 n.p.	No	26	94 (3.2)
Cabanes *et al.* [6]	1993	No	<55 (40.2)	100	43 n.p.	No	56.3	50 (18)
Ranoux *et al.* [5]	1993	No	<55 (38.6)	68	47 n.p.	No	57	No
Schminke *et al.* [12]	1995	Yes	18-87 (57)	100	37 39	Yes	55	No
Molins *et al.* [10]	1996	No	< 45	58	19* 34.5	No	42.8	No
Di Tullio *et al.* [4]	1992	No	(61.4+/-15.7)	146	18* n.p.	No	42	No
Klötzsch *et al.* [8]	1994	No	(61.4+/-15.7)	111	45 46	No	77.5	No
Anzola *et al.* [11]	1995	No	18-75 (45+/-14)	72	52.5 47.5	No	n.d.	No
Job *et al.* [9]	1994	No	< 45	74	51.3 47.3	Yes	66	63 (43)
Jones *et al.* [13]	1994	Yes	(66±13)	220	16 n.p.	No	20	202 (15)
Petty *et al.* [14]	1997	No	(60±3)	116	32 n.p.	No	40	No
Homma *et al.* [20]	1994	No	n.d.	74	31 n.p.	Yes	44	No
Nighoghossian [15]	1996	No	23-59 (47+/-8.9)	118	25.4 n.p.	No	34	No
Serena [21]	1998	Yes	33-85 (64.8+/-12)	208	37.2† 33.5	Yes	56.6	100 (28.2)

n.p.= not performed.

* Used only transthoracic echocardiography.

† Included only cryptogenic stroke.

**Table II:** Annual Risk of Stroke Recurrence in Cryptogenic Stroke with or Without PFO. Prospective Studies

	n (%)	PFO +	PFO -	OR (CI 95%)	Follow-up (months)	Treatment		OR (CI 95%)
							
						AAS	Warfarin	
**Bogousslavsky J *et al*., 1996 [47]**	140 (100)	2·4	---	---	36 (10-91)	No differences		

**De Castro S *et al*., 2000 [36]**	160 (46·3)	3·7	4·5	0·74 (0·30-1·81)	31 (4-58)	Not evaluated		---
Low risk PFO subgroup		1·4						
High risk PFO subgroup		4·2						

**Mass JL *et al*., 2001 [48]**	581 (37)	1·5	1·8	0·90 (0·46-1·82)	37·8 (9·7)	1·6	---	---
PFO+ASA subgroup		3·8		2·98 (1·17-7·58)				

**Homma S *et al*., 2002 [49]**	265 (39)	7·15	6·35	1·14 (0·60-2·17)	24	9	4·8	0·52 (0·16-1·67)
PICSS-Cryptogenic patients								

**Serena J *et al*., 2008 [50]**	486 (41·2)	2·5	3·2	0·87 (0·39-1·93)	24·3 (13·7)	3·5	3·2	0·87 (0·39-1·93)
CODICIA Study									

**Prospective studies in healthy population**								

**Meissner I *et al*, 2006 [51]**	585							
PFO alone	140 (24·3)	2·14	2·17	1·46 (0.74-2.88)	61·2			
PFO+ASA	6 (1·02)	0		3·72 (0·88-15·75)				
ASA alone	5 (1·1)	2 patients						

**Di Tullio M *et al*., 2007 [52]**	1,100	1·22	0·89		79·7 (28·0)			
PFO alone	164 (14·9)			1·64 (0·87-3·09)				
PFO+ASA	27 (2·7)			1·04, (0.14-7.74)				
ASA alone	8 (0·73)			3·66, (0.88-15.30)				

The total number of cryptogenic strokes in the series, the percentage of PFO (between brackets), the time of follow-up as mean (SD) or median [quartiles] as appropriate.
